# Identification of novel helper epitopes of MAGE-A4 tumour antigen: useful tool for the propagation of Th1 cells

**DOI:** 10.1038/sj.bjc.6604966

**Published:** 2009-03-10

**Authors:** T Ohkuri, D Wakita, K Chamoto, Y Togashi, H Kitamura, T Nishimura

**Affiliations:** 1 Division of Immunoregulation, Section of Disease Control, Institute for Genetic Medicine, Hokkaido University, Sapporo, Japan; 2 Division of ROYCE′ Health Bioscience, Institute for Genetic Medicine, Hokkaido University, Sapporo, Japan; 3 Bioimmulance Co. Ltd, Sapporo, Japan

**Keywords:** helper epitope, promiscuous peptide, tumour-specific CD4^+^ T cells, adoptive Th1-cell therapy

## Abstract

MAGE-A4 has been considered as an attractive cancer-testis (CT) antigen for tumour immunotherapy. It has been well accepted that T-helper type 1 (Th1) cell-dominant immunity is critical for the successful induction of antitumour immunity in a tumour-bearing host. The adoptive Th1 cell therapy has been shown to be an attractive strategy for inducing tumour eradication in mouse systems. However, Th1-cell therapy using human tumour-specific Th1 cells, which were expanded from peripheral blood mononuclear cells (PBMCs) in a clinically useful protocol, has never been performed. Here, we first identified MAGE-A4-derived promiscuous helper epitope, peptide (MAGE-A4 280–299), bound to both HLA-DPB1^*^0501 and DRB1^*^1403. Using the peptide, we established a suitable protocol for the propagation of MAGE-A4-specific Th1 cells *in vitro*. Culture of CD4^+^ T cells with IFN-*γ*-treated PBMC-derived adherent cells in the presence of helper epitope peptide resulted in a great expansion of MAGE-A4-reactive Th cells producing IFN-*γ* , but not IL-4. Moreover, it was shown that ligation of MAGE-A4-reactive Th1 cells with the cognate peptide caused the production of IFN-*γ* and IL-2. Thus, our identified MAGE-A4 helper epitope peptide will become a good tool for the propagation of tumour-specific Th1 cells applicable to adoptive immunotherapy of human cancer.

Cancer-testis (CT) antigens can be classified into several super families. MAGE gene group is one of the well-characterised members of the CT antigen family that includes six subfamilies, *MAGE-A*, *MAGE-B*, *MAGE-C*, *MAGE-D*, *MAGE-E* and *MAGE-F*. All of these genes are located on chromosome X. Among these, MAGE-A, MAGE-B and MAGE-C show expression patterns as CT antigens (Van der Bruggen *et al*, 1991; De Plaen *et al*, 1994; Chomez *et al*, 2001). The expression of MAGE-A4 antigen in tumour has been reported in various human malignancies, ovarian neoplasms (Yakirevich *et al*, 2003), mucosal melanomas of the head and neck (Prasad *et al*, 2004), oesophageal adenocarcinomas (Lin *et al*, 2004), colorectal cancer (Li *et al*, 2005) and lung cancer (Tajima *et al*, 2003). Therefore, MAGE-A4 is one of the attractive target molecules in tumour immunotherapy.

Previously, we have proposed that tumour-specific CD4^+^ T cells, especially T-helper type 1 (Th1) cells play a critical role for inducing cytotoxic T lymphocytes (CTL)-mediated antitumour immunity in tumour-bearing mice (Nishimura *et al*, 1999; Ikeda *et al*, 2004; Chamoto *et al*, 2006; Zhang *et al*, 2006). Although many investigators have centred in the activation and targeting of tumour-specific CD8^+^ CTL and CD4^+^ T cells are required for the priming and maintenance of CD8^+^ T cells (Janssen *et al*, 2003; Smith *et al*, 2004; Sun *et al*, 2004). CD4^+^ T cells stimulate CD8^+^ T cells through activating antigen-presenting cells (APCs), such as dendritic cells (DCs) through CD40-CD154 interaction (Bennett *et al*, 1998; Schoenbergner *et al*, 1998). It was also reported that CD4^+^ T cells could stimulate CD8^+^ T cells by their cell–cell communication through cytokines or the direct cell-to-cell interaction (Lu *et al*, 2000; Giuntoli *et al*, 2002). Therefore, fully activated tumour-specific CD4^+^ T cells are important for inducing an effective immunotherapy of the tumour-bearing hosts. For this purpose, many helper epitope peptides have been identified from various tumour antigens (Kobayashi and Celis, 2008). However, there are no reports about helper epitope peptides in MAGE-A4 molecules though several CTL epitopes have been identified (Zhang *et al*, 2002; Miyahara *et al*, 2005). Thus, we planned to identify MAGE-A4-derived helper peptide, which will become a great tool for MAGE-A4-targeted immunotherapy.

Adoptive T-cell therapy is one of the most attractive strategies for inducing tumour eradication. Adoptive immunotherapy using tumour-specific CD8^+^ T cells isolated from resected tumour tissue was carried out for long terms, but no good clinical responses were obtained from these studies (Dudley *et al*, 2001, 2002b). However, recently, adoptive cell transfer of CD8^+^ T cells contaminated with CD4^+^ T cells into lymphodepleting tumour-patients caused a great inhibition of tumour growth (Dudley *et al*, 2002a, 2005). In that therapy, the investigators reported that the existence of CD4^+^ T cells appeared to be important for inducing an efficient therapeutic effect. Moreover, it was shown that adoptive cell transfer of NY-ESO-1-reactive CD4^+^ T cells into melanoma patient resulted in a great inhibition of the growth of metastatic tumour tissue (Hunder *et al*, 2008). We initially showed the crucial role of tumour-specific Th1 cells and the cell transfer of tumour-specific Th1 cells induced tumour-specific CTL *in vivo* to eradicate the established tumour mass (Nishimura *et al*, 1999; Chamoto *et al*, 2003). This result raised the possibility that Th1 cell therapy will become a good strategy for the treatment of human cancer.

Here, we identified new MAGE-A4-derived helper peptides efficiently presented by APC. The one of the peptides binds to both HLA-DPB1^*^0501 and DRB1^*^1403, indicating that it is a promiscuous peptide. Using the peptide, we established a suitable protocol for the propagation of MAGE-A4-specific CD4^+^ T cells applicable to adoptive Th1-cell therapy of human cancer. Moreover, we showed that helper epitope peptide is a good tool for the activation of MAGE-A4-specific Th1 cells to produce Th1 cytokines, which will preferable to activate CTL-mediated antitumour immunity. Thus, we believe that our identified MAGE-A4-specific CD4-helper epitope peptides will become a good tool for the application to the therapy of human cancer.

## Materials and methods

### Cell lines and culture medium

Epstein–Barr virus-transformed B cells (EBV-B) were generated from peripheral blood mononuclear cells (PBMCs) of healthy volunteers by culturing with culture supernatant from the EBV-producing B95-8 cell line after obtaining written informed consent approved by medical ethics committees of Hokkaido University Graduate School of Medicine. These cells were maintained in RPMI 1640 (Sigma, St Louis, MO, USA) containing 10% heat-inactivated foetal bovine serum (Life Technologies Inc., Invitrogen, Carlsbad, CA, USA) with 2 mM L-glutamine, 0.05 mM 2-mercaptoethanol (Sigma), 10 mM HEPES, 100 U ml^−1^ penicillin, and 100 *μ*g ml^−1^ streptomycin sulphate. Dendritic cells (DC) and T cells were derived from PBMC of healthy volunteers after obtaining written informed consent. Dendritic cells were cultured in AIM-V (Life Technologies) medium without serum. T cells were cultured in AIM-V medium containing 5% heat-inactivated pooled human AB serum (kindly donated from Hokkaido red cross blood centre) or 5% heat-inactivated foetal bovine serum (Life Technologies).

### Peptides, recombinant protein

Synthetic peptides (purity>95%) were purchased from Greiner Bio-One (Tokyo, Japan). MAGE-A4 overlapping peptides were purchased from SIGMA genosys (Hokkaido, Japan). The 44 overlapping peptides covering whole amino-acid sequence of MAGE-A4 were synthesised and designated as M1-M44 peptide (M1-M43: 20-mer peptide, M44: 17-mer peptide). For the initial screening assay, these 44 peptides were subdivided into MIX1-MIX9 peptides pools (MIX1; M1-M5, MIX2; M6-M10, MIX3; M11-M15, MIX4; M16-M20, MIX5; M21-M25, MIX6; M26-M30, MIX7; M31-M35, MIX8; M36-M40, and MIX9; and M41-M44). Recombinant MAGE-A4 protein was expressed in *Escherichia coli* as full-length protein.

### Preparation of PBMC-derived dendritic cells as antigen-presenting cells

Peripheral blood mononuclear cells from healthy volunteers were isolated by Ficoll-Paque (Amersham Bioscience, Uppsala, Sweden) gradient centrifugation. Peripheral blood mononuclear cells were incubated in 6-well plate (Nalge Nunc International, Roskilde, Denmark) in AIM-V medium without serum. Non-adherent cells were removed and adherent cells were cultured in the presence of GM-CSF (30 ng ml^−1^; KIRIN, Tokyo, Japan) and IL-3 (30 ng ml^−1^; KIRIN) in AIM-V (Sato *et al*, 1999; Buelens *et al*, 2002). Medium was exchanged with fresh medium containing GM-CSF and IL-3 on days 3. On day 7, the adherent DCs were harvested with trypsine (Sigma) and used as APC. The expression of CD11c was >99% for these DCs (data not shown).

### *In vitro* stimulation of helper T cells with recombinant protein-pulsed DC

CD4^+^ T cells were purified from PBMC by EasySep CD4 T-cell positive selection kit (Veritas, Tokyo, Japan). Dendritic cells, which were generated from adherent PBMCs by culture with GM-CSF, and IL-3 *in vitro* were pulsed with 50 *μ*g ml^−1^ rMAGE-A4 protein for 2 h. CD4^+^ T cells (1 × 10^6^) were stimulated with autologous mitomycin C (MMC)–treated antigen-pulsed DC (1 × 10^5^) in 24-well culture plate (BD Biosciences, San Jose, CA, USA) in AIM-V medium supplemented with 5% human AB serum. Seven days later, the stimulated CD4^+^ T cells were restimulated with MMC-treated autologous DC pulsed with rMAGE-A4 protein (50 *μ*g ml^−1^). Two days after the secondary stimulation with antigen, human recombinant IL-2 (kindly supplied by Shionogi Pharmaceutical Institute Co. Ltd, Osaka, Japan) was added at a final concentration of 10 IU ml^−1^. One week later, the T cells were divided into nine cultures, and each culture was stimulated with PBMC pulsed with peptides mixture (MIX1-MIX9), including MAGE-A4-derived overlapping peptides. After stimulation with peptides mixture, the T-cell cultures were tested for their cytokine production in response to peptides mixture. The T cells showing a specific response to the cognate mixture were expanded and used for further analysis.

### Antigen-specific cytokine production of T cells

T cells (3 × 10^4^ per well) were cultured with or without MMC-treated autologous PBMC (3 × 10^4^ per well) or EBV-B cells (1 × 10^4^ per well) in the presence of relevant antigen in 96-well culture plate (BD Biosciences). After 20 h, culture supernatants were collected and cytokine (IFN-*γ*, IL-4, or IL-2) level was measured by ELISA kit (BD Biosciences). Major histocompatibility complex (MHC)-restriction was determined by adding anti-HLA-DR monoclonal antibody (mAb) (L243; BD Bioscience), anti-HLA-DP mAb (BRAFB6), or anti-HLA-DQ mAb (SPV-L3; Serotech Ltd, Raleigh, NC, USA). All mAbs were used at a final concentration of 5 *μ*g ml^−1^.

### Establishment of the protocol for the induction of MAGE-A4-specific Th cells useful for clinical therapy

Isolated PBMC was incubated in 24-well culture plate in serum-free AIM-V medium with 20 ng ml^−1^ IFN-*γ* (PeproTech, Inc, Rocky Hill, NJ, USA). After 2 h, the adherent PBMCs were treated with MMC. The isolated CD4^+^ T cells were cultured with IFN-*γ*-treated PBMC in the presence of MAGE-A4 peptide (10 *μ*g ml^−1^). Seven days later, the cultured CD4^+^ T cells were restimulated with IFN-*γ*-treated adherent cells derived from PBMC. Further seven days later, peptide-pulsed PBMCs were used as APC to expand peptide-specific Th cells. The expression of cell surface markers of IFN-*γ*-treated PBMC was determined by staining with fluoresceinisothiocyanate (FITC)-conjugated mouse anti-HLA-A/B/C mAb, FITC-conjugated mouse anti-HLA-DR/DP/DQ mAb, FITC-conjugated mouse anti-human CD86 mAb, or FITC-conjugated mouse isotype control mAb (BD Biosciences). The cell-associated fluorescence was determined by a multicolour flow cytometer (FACSCalibar, BD Biosciences) and analysed with a computer software (CellQuest, BD Biosciences).

### Measurement of cytokine production by Intracellular staining assay

The cultured Th cells were stimulated with autologous EBV-B cell lines in the presence of cognate or irrelevant peptide, respectively. After the 2-h incubation, brefeldin-A (Sigma) was added to the samples and the cells were incubated for an additional 4 h. The intracellular cytokine levels of the cells were determined by staining with R-phycoerythrin (PE)/cyanine-conjugated mouse anti-human CD4 mAb and PE-conjugated mouse anti-human IL-4 mAb and FITC-conjugated mouse anti-human IFN-*γ* mAb or PE-conjugated mouse anti-human CD8 mAb. These antibodies were purchased from BD Biosciences. The cell-associated fluorescence was determined by a multicolour flow cytometer (FACSCalibar, BD Biosciences) and analysed with a computer software (CellQuest, BD Biosciences).

## Results

### Identification of MAGE-A4-derived helper epitopes

**Figure 1:**
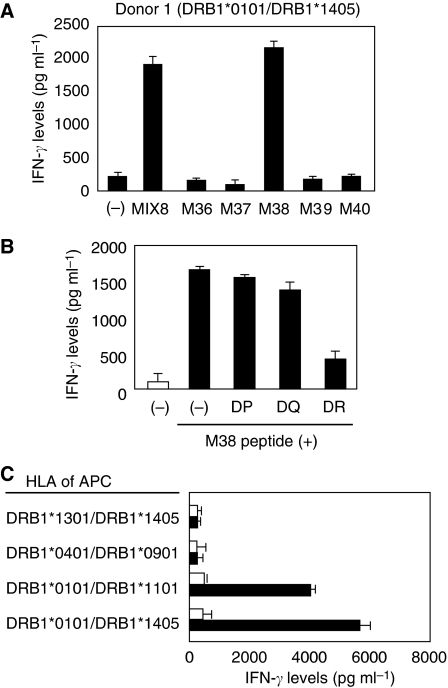
Induction of HLA-DRB1^*^0101-restricted MAGE-A4-specific CD4^+^ T cells from donor 1. (**A**) Donor 1-derived CD4^+^ T cells were assessed the specificity to overlapping peptides and their mixture, MIX8. The CD4^+^ T cells were stimulated with autologous PBMC alone (−) or PBMC pulsed with MIX8 (10 *μ*g ml^−1^) or with each of overlapping peptide in the MIX8 (10 *μ*g ml^−1^). HLA types of the donors were displayed over the panel. (**B**) M38-specific Th cells were stimulated with autologous PBMC pulsed with irrelevant (open bar) or cognate (filled bar) peptide in the presence of 5 *μ*g ml^−1^ of mAbs against HLA-DP, HLA-DQ or HLA-DR. (**C**) M38-specific Th cells were stimulated with allogeneic EBV-B cell lines pulsed with irrelevant (open bar) or cognate (filled bar) peptide. All T-cell cultures were incubated with the MMC-treated target cells in duplicate for 20–24 h, then the culture supernatants were assessed for IFN-*γ* levels in ELISA. These experiments were carried out independently at least three times. Data denote mean±s.e.m. and are representative of the experiments.

To identify MAGE-A4-derived helper epitopes, purified CD4^+^ T cells from two healthy donors were stimulated with autologous monocyte-derived DC pulsed with rMAGE-A4 protein. Then, specific responses of the cultured CD4^+^ Th cells to MAGE-A4-derived peptide were examined in ELISA. In donor 1, one Th cell line produced significant IFN-*γ* against autologous PBMCs pulsed with mixture of MAGE-A4-derived overlapping peptide. Moreover, it was shown that Th cells produced IFN-*γ* in response to M38 peptide included in MIX8 (Figure 1A). To elucidate HLA-restriction of the Th cell line, we used mAb against HLA-DP, HLA-DQ and HLA-DR. The IFN-*γ* production of Th cell line against M38 peptide was significantly reduced when mAb against HLA-DR was added, whereas mAbs against HLA-DP or HLA-DQ had no effect (Figure 1B). To further analyse the HLA-restriction, we tested the reactivity of Th cells against a range of peptide-pulsed allogeneic EBV-B cell lines with known HLA-DR genotypes. As shown in Figure 1C, the Th cells recognised only HLA-DRB1^*^0101-expressing EBV-B cell lines, suggested that this M38 peptide was presented by HLA-DRB1^*^0101.

**Figure 2:**
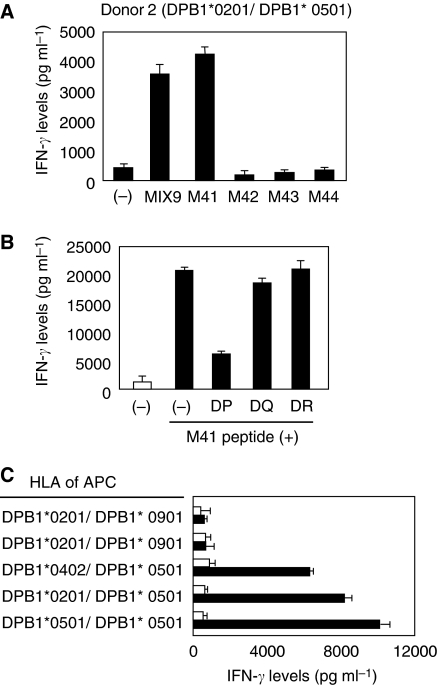
Induction of HLA-DPB1^*^0501-restricted MAGE-A4-specific CD4^+^ T cells from donor 2. (**A**) Donor 2-derived CD4^+^ T cells were assessed the specificity to overlapping peptides and their mixture, MIX9. The CD4^+^ T cells were stimulated with autologous PBMC alone (−) or PBMC pulsed with MIX9 (10 *μ*g ml^−1^) or with each of overlapping peptide in the MIX9 (10 *μ*g ml^−1^). HLA types of the donor were displayed over the panel. (**B**) M41-specific Th cells were stimulated with autologous PBMC pulsed with irrelevant (open bar) or cognate (filled bar) peptide in the presence of 5 *μ*g ml^−1^ of mAbs against HLA-DP, HLA-DQ or HLA-DR. (**C**) M41-specific Th cells were stimulated with allogeneic EBV-B cell lines pulsed with irrelevant (open bar) or cognate (filled bar) peptide. All T-cell cultures were incubated with the MMC-treated target cells in duplicate for 20–24 h, then the culture supernatants were assessed for IFN-*γ* levels in ELISA. These experiments were carried out independently at least three times. Data denote mean±s.e.m. and are representative of the experiments.

**Figure 3:**
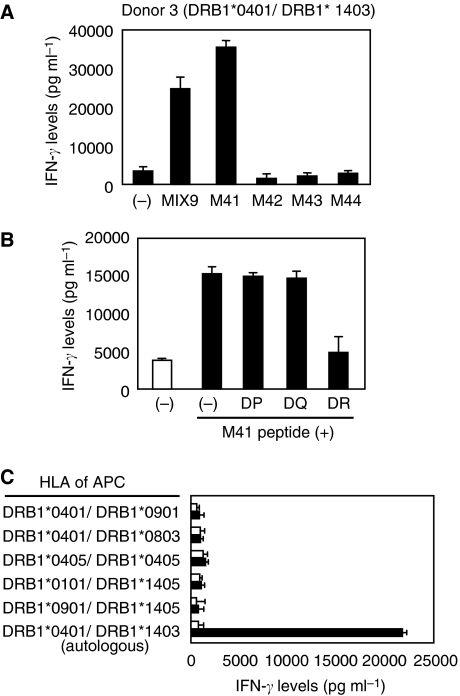
Induction of HLA-DRB1^*^1403-restricted MAGE-A4-specific CD4^+^ T cells from donor 3. (**A**) Donor 3-derived CD4^+^ T cells were assessed the specificity to overlapping peptides and their mixture, MIX9. The CD4^+^ T cells were stimulated with autologous PBMC alone (−) or PBMC pulsed with MIX9 (10 *μ*g ml^−1^) or with each of overlapping peptide in the MIX9 (10 *μ*g ml^−1^). HLA types of the donor were displayed over the panel. (**B**) M41-specific Th cells were stimulated with autologous PBMCs pulsed with irrelevant (open bar) or cognate (filled bar) peptide in the presence of 5 *μ*g ml^−1^ of mAbs against HLA-DP, HLA-DQ or HLA-DR. (**C**) M41-specific Th cells were stimulated with allogeneic or autologous EBV-B cell lines pulsed with irrelevant (open bar) or cognate (filled bar) peptide. All T-cell cultures were incubated with the MMC-treated target cells in duplicate for 20–24 h, then the culture supernatants were assessed for IFN-*γ* levels in ELISA. These experiments were carried out independently at least three times. Data denote mean±s.e.m. and are representative of the experiments.

CD4^+^ Th cells derived from donor 2 revealed the specific IFN-*γ* production in response to autologous PBMC pulsed with M41 peptide (MAGE-A4; AETSYVKVLEHVVRVNARVR) in MIX9. The response was reduced in the presence of anti-HLA-DP mAb, but not HLA-DQ nor HLA-DR (Figure 2B). The Th cells recognised only HLA-DPB1^*^0501-expressing EBV-B cell lines pulsed with the M41 peptide, suggested that the M41 peptide was presented by HLA-DPB1^*^0501 (Figure 2C). As this allele is expressed in more than half of the numbers of Japanese, the M41 peptide is very useful to generate MAGE-A4-specific helper T cells from many patients. Moreover, M41 peptide-specific Th cell line was also obtained from donor 3 (Figure 3A). Unlike the case of the donor 2, this recognition of the CD4^+^ T cells was inhibited by anti-HLA-DR mAb (Figure 3B). Because HLA-DR genotypes of donor 3 are DRB1^*^0401 and DRB1^*^1403, we attempted to use allogeneic EBV-B cell lines expressing matched genotype. However, we failed to find any DRB1^*^1403-expressing cell lines. Therefore, we used DRB1^*^1405-expressing cell lines instead of the DRB1^*^1403-cell lines. Consequently, the donor 3-derived Th cells showed a specific reaction to M41 peptide against only autologous cell line, but not DRB1^*^0401-expressing allogeneic cell lines (Figure 3C). Thus, the M41 peptide has capability of binding to HLA-DRB1^*^1403, suggesting that the M41 peptide is promiscuous and would be available with tumour immunotherapy. Therefore, we focused on this peptide in the further experiments.

### Identifying minimal epitopes of MAGE-A4 recognised by the CD4^+^ T-cell lines

**Figure 4:**
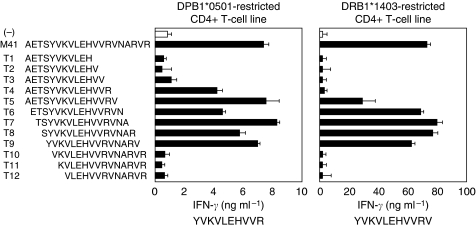
Identification of minimal epitopes recognised by two M41 peptide-specific CD4^+^ T-cell lines. Two M41 peptide-specific CD4^+^ T-cell lines derived from donors 2 and 3 were cultured with their autologous PBMCs pulsed with a series of 15-mer overlapping peptides encompassing M41 peptide (MAGE-A4 280–299). The left panel showed reactivity of HLA-DRB1^*^0501-restricted CD4^+^ T-cell lines and the right panel showed HLA-DRB1^*^1403-CD4^+^ T-cell lines. Each minimal epitope of two cell lines were displayed under their graphs. Each T-cell line was stimulated with the MMC-treated target cells pulsed with the indicated peptide (10 *μ*g ml^−1^) in duplicate for 20 h, then the culture supernatants were assessed for IFN-*γ* levels in ELISA. These experiments were carried out independently at least three times. Data denote mean±s.e.m. and are representative of the experiments.

To identify MAGE-A4 minimal epitopes recognised by M41-specific CD4^+^ T-cell lines from donor 2 and donor 3, respectively, a series of 15-mer overlapping peptides derived from M41 peptide were prepared and tested for their ability to stimulate IFN-*γ* release from the both T-cell lines. As shown in Figure 4, the HLA-DPB1^*^0501-restricted CD4^+^ T cells recognised EBV-B cells in the presence of p4 to p9 peptides, suggesting that this minimal region was MAGE-A4 284–293 (YVKVLEHVVR; Figure 4, left panel). In contrast, the HLA-DRB1^*^1403-restrected CD4^+^ T cells recognised EBV-B cells in the presence of p5 to p9 peptides, suggesting that this minimal region was MAGE-A4 284–294 (YVKVLEHVVRV; Figure 4, right panel). Thus, there was a little difference in the epitope recognised by two CD4^+^ T-cell lines.

### Generation of MAGE-A4-specific CD4^+^ T cells by using a synthetic peptide

**Figure 5:**
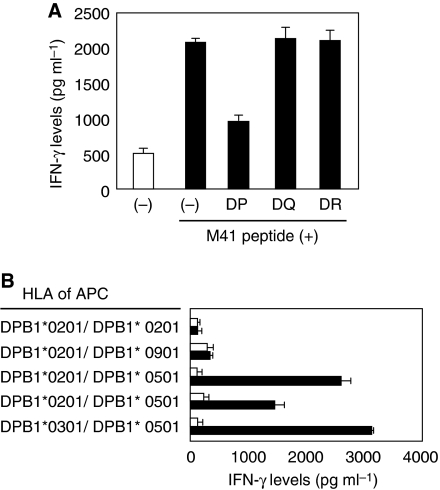
Induction of HLA-DPB1^*^0501-restricted MAGE-A4-specific CD4^+^ T cells from donor 4 using a synthetic M41 peptide. (**A**) Donor 4-derived CD4^+^ T cells were primed with autologous DC pulsed with synthetic M41 peptide (10 *μ*g ml^−1^), but not rMAGE-A4 protein. The primed CD4^+^ T cells were stimulated with autologous PBMC pulsed with irrelevant peptide (open bar) or cognate peptide (filled bar) in the presence of 5 *μ*g ml^−1^ of mAbs against HLA-DP, HLA-DQ or HLA-DR. (**B**) The M41-specific Th cells were stimulated with allogeneic EBV-B-cell lines pulsed with irrelevant (open bar) or cognate (filled bar) peptide. All T-cell cultures were incubated with the MMC-treated target cells in duplicate for 20–24 h, then the culture supernatants were assessed for IFN-*γ* levels in ELISA. These experiments were carried out independently at least three times. Data denote mean±s.e.m. and are representative of the experiments.

We assessed whether a synthetic M41 peptide is able to generate MAGE-A4-specific CD4^+^ T cells. CD4^+^ T cells derived from a donor 4 with HLA-DPB1^*^0501 were stimulated with their autologous monocyte-derived DC pulsed with a synthetic M41 peptide. As a result of repetitive stimulation with the peptide, the CD4^+^ T cells produced IFN-*γ* in response to the M41 peptide bound to HLA-DPB1^*^0501 molecule in APC (Figure 5A and B), indicating that the synthetic M41 peptide is able to induce MAGE-A4-specific CD4^+^ T cells effectively.

### Development of a protocol for the inducing and propagating peptide-specific Th1 cells applicable to adaptive immunotherapy

**Figure 6:**
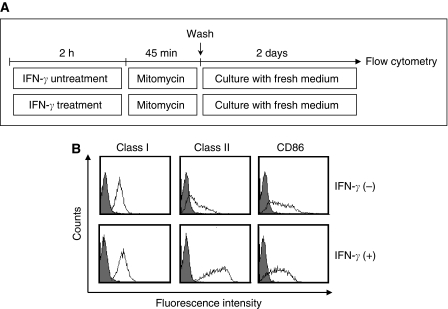
IFN-*γ* treatment upregulated the expression of cell surface molecules on PBMC-derived adherent cells. (**A**) PBMC were incubated in the absence or presence of IFN-*γ* (20 ng ml^−1^) for 2 h. After treatment of MMC, the adherent cells were incubated in fresh culture medium for 2 days. (**B**) For analysis of the expression of cell surface molecules on the cells, the cells were stained with anti-HLA class I, anti-HLA DP/DQ/DR, anti-human CD86 or control antibody for 15 min, and analysed in a flow cytometry. These experiments were carried out independently at least three times. Data are representative of the experiments.

**Figure 7:**
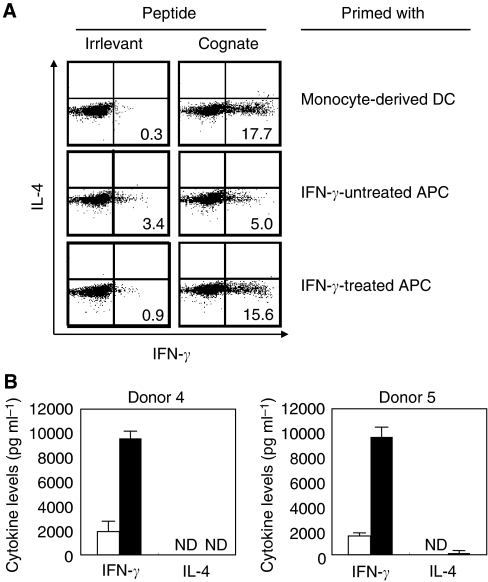
Efficient inductions of MAGE-A4-specific CD4^+^ T cells primed with IFN-*γ*-treated adherent cells. (**A**) CD4^+^ T cells were primed with monocyte-derived DC, IFN-*γ*-untreated APC or IFN-*γ*-treated APC in the presence of 10 *μ*g ml^−1^ M41 peptide. Specific responses of each CD4^+^ T cells were assessed in an intracellular staining assay with PE-conjugated anti-IL-4 mAb and FITC-conjugated anti-IFN-*γ* mAb. Numbers represents percentages of IFN-*γ*-producing cells in CD4^+^ T cells. Data are representative of three independent experiments. (**B**) CD4^+^ T cells from donors 4 and 5 primed with IFN-*γ*-treated adherent cells were assessed their cytokine production profiles in ELISA. ND=not detected. These experiments were carried out independently at least three times. Data denote mean±s.e.m. and are representative of the experiments.

**Figure 8:**
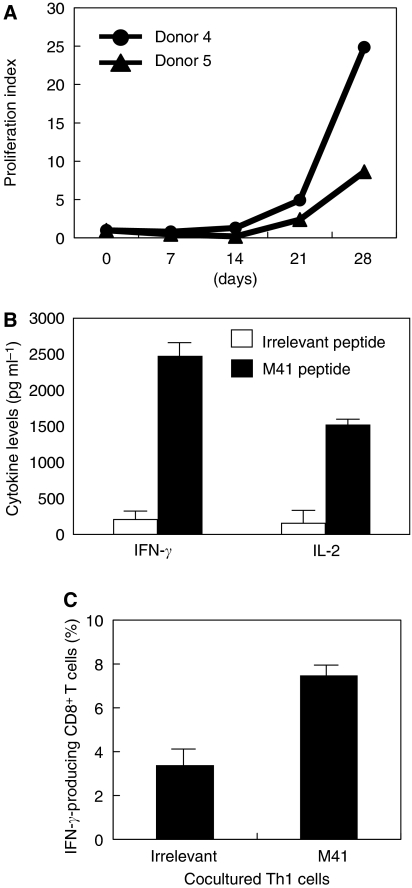
The number of MAGE-A4-specific Th1 cells increased *in vitro* culture and the expanded Th1 cells were activated in the presence of the cognate peptide without APC. (**A**) CD4^+^ T cells from donors 4 and 5 were stimulated with APC pulsed with MAGE-A4-helper epitope peptide weekly. At days 0, 7, 14, 21 and 28, the cultured T cells were collected and the total cell number was counted. The growth rates compared with number at day 0 were displayed. (**B**) In the absence of APC, MAGE-A4-speficific Th1 cells were cultured with irrelevant (open bar) or cognate (filled bar) peptide (10 *μ*g ml^−1^) for 24 h. The culture supernatants were collected and assessed cytokine levels in ELISA. (**C**) In the absence of APC, the Th1 cells were cultured with autologous CD8^+^ T cells in the presence of control (irrelevant) or cognate (M41) peptide (10 *μ*g ml^−1^). At day 5, IFN-*γ*-producing CD8^+^ T cells in stimulating with anti-CD3 mAb were assessed by intracellular staining using PE-conjugated anti-CD8 mAb and FITC-conjugated anti- IFN-*γ* mAb. These experiments were carried out independently at least three times. Data denote mean±s.e.m. and are representative of the experiments.

To apply our identified helper epitope peptide to the clinical trial of Th1-cell therapy, we tried to establish a protocol for the induction and propagation of MAGE-A4 peptide-specific Th1 cells useful for Th1 cell therapy. In general, monocyte-derived DC expanded *in vitro* are used as APC for stimulating T cells with peptide. However, it takes approximately 7 days to prepare DC from PBMC. Therefore, we developed an alternative method. We used freshly isolated PBMC-derived adherent cells as a source of APC. The detailed protocol for preparing APC was shown in Figure 6A. IFN-*γ*-treated adherent cells upregulated its expression of HLA-class I, HLA-class II and CD86, respectively, compared with freshly isolated adherent cells (Figure 6B). As shown in Table 1, the same results were obtained in two donors. Thus, we evaluated whether IFN-*γ*-treated adherent cells derived from PBMC had a capability of initiating peptide-induced stimulation for enhancing the propagation of MAGE-A4 peptide-specific Th1 cells. CD4^+^ T cells from PBMC of donor 4 and donor 5 were primed by each autologous APC in the presence of M41 peptide. As shown in Figure 7A, peptide-specific CD4^+^ T cells were generated more efficiently when IFN-*γ*-treated adherent cells were used as APC, compared with the case that freshly isolated adherent cells were used. Surprisingly, the frequency of peptide-specific CD4^+^ T cells primed with IFN-*γ*-treated adherent cells was almost the same as that primed with monocyte-derived DC. The generated Th cells produced much IFN-*γ* and less IL-4 in the presence of cognate peptide, indicating peptide-specific Th1 cells were dominantly activated in this condition (Figure 7B). We also showed that repetitive stimulation of Th1 cells with APC pulsed with MAGE-A4 helper epitope peptide resulted in 10-25-fold increase of the numbers of Th1 cells within a 28-day culture (Figure 8A). All the frequencies of helper (CD4-positive) T cells expanded were over 97% (data not shown).

### Activation of MAGE-A4-specific Th1 cells with MAGE-A4 helper epitope peptide in the absence of APC

The expanded CD4^+^ Th1 cells expressed MHC class II molecules and costimulatory molecules on their cell surface. Therefore, we tried whether MAGE-A4 helper epitope peptide could activate Th1 cells to produce IFN-*γ* and IL-2 in the absence of APC. As shown in Figure 8B, addition of M41 peptide, but not irrelevant peptide to the samples resulted in the production of IFN-*γ* and IL-2 from Th1 cells. Moreover, addition of M41 peptide to the mixed culture of Th1 cells and CD8^+^ T cells resulted in the induction of IFN-*γ*-producing CD8^+^ T cells, which is preferable to induce Th1-dependent antitumour immunity (Figure 8C).

## Discussion

Recently, it has been indicated that CD4^+^ Th cells play a crucial role in tumour immunology in addition to CD8^+^ CTL in both animal and human systems (Nishimura *et al*, 1999; Marzo *et al*, 2000; Ikeda *et al*, 2004; Janssen *et al*, 2005; Hunder *et al*, 2008). Indeed, in clinical study, our analysis of specimens from non-small cell lung carcinoma (NSCLC) showed significant correlations between infiltration of both CD8^+^ and CD4^+^ T cells into tumour nest and prognosis (Yoshida *et al*, 2006). Thus, it is important to develop the new immunotherapy, as using HLA class I and II-binding peptide-based vaccine (Odunsi *et al*, 2007) or antigen protein-based vaccine as activating both CD8^+^ and CD4^+^ effector T cells (Atanackovic *et al*, 2004; Uenaka *et al*, 2007). It has been, however, known that many regulatory T cells were induced in tumour-bearing hosts to inhibit effector function of CTL or Th cells (Liyanage *et al*, 2002). Therefore, it is difficult to activate these tumour-specific effector T cells under the suppressive condition in a tumour-bearing host. To overcome the problem, we initially proposed the application of tumour-specific Th1 cells to adoptive tumour immunotherapy (Nishimura *et al*, 1999; Ikeda *et al*, 2004; Chamoto *et al*, 2006; Zhang *et al*, 2006). It has been well accepted that Th1-dominant immunity is critical for the successful induction of antitumour immunity in tumour-bearing hosts (Trinchieri, 2003 ). Our earlier study in animal models showed that the transfer of OVA-specific Th1 eradicated the transplanted OVA-expressing tumour. The spleen cells from mice treated with OVA-specific Th1 cells contained OVA-specific CTL, whereas OVA-specific Th2 cell therapy did not induced functional CTL (Nishimura *et al*, 1999). Moreover, by the combination therapy of OVA-specific TCR gene-modified Th1 cells with cyclophosphamide, OVA-expressing tumour was completely eradicated (Chamoto *et al*, 2004). In addition, in the transfer model of the gene-modified tumour-specific human T cells into immunodeficient mouse, combination treatment with tumour-specific Tc1 and Th1 completely rejected tumour, although tumour-specific Tc1 alone did not show such an effect (Gyobu *et al*, 2004). These results suggest that Th1-cell therapy has the potential of enhancing antitumour immunity in patients with cancer. Therefore, we attempted to develop a protocol for the propagation of tumour-specific human Th1 cells *in vitro*. The choice of targeted tumour antigen is of importance in the conduct of clinical trials of tumour immunotherapy. MAGE-A4 is an attractive antigen because of its wide expression in a variety of tumours and its immunogenicity (Tajima *et al*, 2003; Yakirevich *et al*, 2003; Lin *et al*, 2004; Prasad *et al*, 2004; Li *et al*, 2005). Earlier studies reported the generation of MAGE-A4-specific CTL clones and definition of its epitope peptides (Zhang *et al*, 2002; Miyahara *et al*, 2005). However, no helper epitopes of MAGE-A4 have been reported. In this study, we first identified the helper epitope peptides derived from MAGE-A4, which are presented to DPB1^*^0501 and DRB1^*^1403, indicating that this tumour peptide is a promiscuous peptide (Figure 4). In several tumour antigens, promiscuous peptides have been identified (Kobayashi *et al*, 2000; Ohkuri *et al*, 2007). Binding ability of these peptides to multiple HLA could overcome the limitation of HLA restriction of cancer patients. In addition, as DPB1^*^0501, which M41 peptide binds to, is frequently expressed in Japanese population (approximately 60%), it is expected that our defined helper peptide would contribute to the tumour immunotherapy of patients with MAGE-A4-expressing tumour.

In this study, we established a protocol for the efficient induction and propagation of peptide-specific Th1 cells. In most of adoptive cell therapy so far performed, the injected cells were mainly expanded from TIL by culturing with anti-CD3 mAb and high-dose IL-2 (Dudley *et al*, 2001, 2005). As it is difficult to obtain TIL from each patient, the strategy has a limitation. To overcome this limitation, Morgan *et al* (2006) developed the approach to therapy based on the genetic modification of PBMC. They reported that transfer of tumour antigen-specific TCR gene-modified PBL enhanced antitumour immunity in the patients. Moreover, Labarriere *et al* (2008) recently reported that PBMCs were good sources of CTL as well as TIL. In fact, they had performed adoptive cell therapy in clinical trial, in which PBMC-derived CTL clones were used (Vignard *et al*, 2005). Likewise, our strategy is that PBMCs are used as sources of CD4^+^ T-cell for generating tumour-specific Th cells. In addition, we used PBMC-derived adherent cells as antigen-presenting cells. This strategy has the advantage that it does not take a long time from collection of blood to the initiation of CD4^+^ T-cell sensitisation. Although freshly isolated PBMC-derived adherent cells had less stimulatory activity, IFN-*γ*-treated adherent cells showed the upregulated expression of MHC class II and CD86 costimulatory molecules to acquire stimulatory activity as well as monocyte-derived DC (Figure 6B and Table 1). Moreover, the expanded MAGE-A4-specific Th cells produced high levels of IFN-*γ* and less IL-4 in two healthy donors tested, suggested that the Th cells would differentiate into Th1 cells (Figure 7B). Moreover, the Th cells produced neither IL-10 nor IL-17, and did not detect Foxp3 expression (data not shown). Therefore, our developed method may be beneficial to apply adoptive Th1 cell therapy to clinical study.

Finally, as shown in Figure 8B, we also showed that MAGE-A4 helper epitope peptides were presented to MHC class II molecules on MAGE-A4-specific Th1 cells to stimulate Th1-derived cytokine release, which is preferable to induce IFN-*γ*-producing Tc1 cells essential for eradication of tumour cells.

In conclusion, we first identified MAGE-A4-derived helper peptides, which will become a good tool for propagating and activating MAGE-A4 peptide-specific Th1 cells. These findings would contribute to a clinical trial of adoptive Th1-cell therapy against MAGE-A4-expressing human cancer in future. Recently, Hunder *et al* (2008) reported that NY-ESO-1-specific CD4^+^ T cells into patients with metastatic melanoma caused a great inhibition of tumour. The new challenge to the adoptive immunotherapy is now started and we believed that Th1-cell therapy using tumour-specific Th1 cells expanded with helper epitope peptide would become a promising strategy for the immunotherapy of human cancer.

## Figures and Tables

**Table 1:** IFN-*γ* treatment upregulated the expression of cell surface molecules on PBMC-derived adherent cells

**Cell surface markers**	**HLA class I**		**HLA class II**		**CD86**	
**IFN-***γ* **treatment**	**(−)**	**(+)**	**(−)**	**(+)**	**(−)**	**(+)**
Donor 4	14.6^a^	21.4	41.3	178	17.4	25.2
Donor 5	22.4	37.6	210	688	21.2	35.3

a Figures represent mean fluorescence intensity detected by flow cytometry.
